# Booster Effect of a Natural Extract of *Polypodium leucotomos* (Fernblock®) That Improves the UV Barrier Function and Immune Protection Capability of Sunscreen Formulations

**DOI:** 10.3389/fmed.2021.684665

**Published:** 2021-06-02

**Authors:** Jose Aguilera, Miguel Vicente-Manzanares, María Victoria de Gálvez, Enrique Herrera-Ceballos, Azahara Rodríguez-Luna, Salvador González

**Affiliations:** ^1^Photobiological Dermatology Laboratory, Department of Dermatology and Medicine, Faculty of Medicine, Medical Research Center, University of Málaga, Málaga, Spain; ^2^Molecular Mechanisms Program, Centro de Investigación del Cáncer and Instituto de Biología Molecular y Celular del Cáncer, Consejo Superior de Investigaciones Científicas-University of Salamanca, Salamanca, Spain; ^3^Dermatology Service, Hospital Clínico Universitario Virgen de la Victoria, Málaga, Spain; ^4^Innovation and Development, Cantabria Labs, Madrid, Spain; ^5^Department of Medicine and Medical Specialties, Alcalá de Henares University, Madrid, Spain

**Keywords:** ultraviolet radiation, sunscreens, *Polypodium leucotomos* extract, booster effect, human immunoprotection factor, sun protection factor, UVA protection factor

## Abstract

**Background:** Novel approaches to photoprotection must go beyond classical MED measurements, as discoveries on the effect of UV radiation on skin paints a more complex and multi-pronged scenario with multitude of skin cell types involved. Of these, photoimmunoprotection emerges as a crucial factor that protects against skin cancer and photoaging. A novel immune parameter is enabled by the precise knowledge of the wavelength and dose of solar radiation that induces photoimmunosupression. Natural substances, that can play different roles in photoprotection as antioxidant, immune regulation, and DNA protection as well as its possible ability as sunscreen are the new goals in cosmetic industry.

**Objective:** To analyze the effect of a specific natural extract from *Polypodium leucotomos* (PLE, Fernblock®), as part of topical sunscreen formulations to protect from photoimmunosuppression, as well as other deleterious biological effects of UV radiation.

**Methods:** The possible sunscreen effect of PLE was analyzed by including 1% (w/w) PLE in four different galenic formulations containing different combinations of UVB and UVA organic and mineral filters. *In vitro* sun protection factor (SPF), UVA protection factor (UVA-PF), contact hypersensitivity factor (CHS), and human immunoprotection factor (HIF) were estimated following the same protocol as ISO 24443:2012 for in vitro UVA-PF determination.

**Results:** PLE-containing formulations significantly reduced UV radiation reaching to skin. Combination of UVB and UVA filters with PLE increased SPF and UVAPF significantly. PLE also increased UV immune protection, by elevating the contact hypersensitivity factor and the human immunoprotective factor of the sunscreen formulations.

**Conclusion:** This study confirms the double role of PLE in photoprotection. Together to the biological activity shown in previous works, the UV absorption properties of PLE confers a booster effect when it is supplemented in topical sunscreens increasing the protection not only at level of erythema and permanent pigment darkening but also against two photoimmunoprotection factors.

## Introduction

The skin is the first barrier of the organism against aggression. Biological aggression usually brings to mind pathogens, e.g., viruses or bacteria. However, the skin also protects from mechanical and radiation damage. The latter is crucial due to the constant irradiation of the Earth's surface with sun rays, which contain a significant amount of UV photons. UV radiation comprises photons from ~100 to 400 nm in wavelength, of which those between 290 and 400 nm have significant biological effects at earth surface. Although some effects on human skin are beneficial [for example, vitamin D synthesis (1)], most are deleterious. Short-term deleterious effects are sunburn, oxidative stress as well as skin pigmentation changes leading in the long-term an increase in photoaging damage as well as the probability of photocarcinogenesis. Sunburn refers to the destruction of epidermal tissue, and includes redness and swelling, blood vessel dilation and inflammation. These processes are collectively known as erythema. Photoaging refers to the inability of the skin to recover its mechanical properties (particularly elasticity) after sun exposure, and it is related to increased metalloprotease and elastase secretion (2), and an overall decrease in the ability of the skin to locally replenish sunburnt populations (3). Finally, photocarcinogenesis refers to the malignant transformation that UV radiation may cause on skin cells, either by direct DNA mutation (mainly formation of T-T dimers) or by indirect means [oxidative damage to the DNA, recently reviewed in Lee et al. (4)].

Since the beginning of the development of skin photoprotection, prevention of the generation erythema is the most extended indicator when measuring the efficacy of photoprotective measures, particularly sunscreens. Different international organizations, including The American Food and Drug Administration (FDA) or European Cosmetics Agency have provided guidelines that control the efficacy of sunscreens by means of *in vivo* and *in vitro* methods, that are finally described in the standards ISO 24444:2019 and the ISO 24443:2012 respectively. Although the European regulatory body (EMA) classifies sunscreens as cosmetic products [Regulation (EC) No 1223/2009], it does require the manufacturer to provide truthful and useful information regarding its use [Regulation (EU) No 655/2013], which, in practical terms, enforces the use of SPF or a similar parameter.

The aforementioned regulations do, in fact, enforce the SPF as the single standardized regulatory element that controls the efficacy and marketability of a given sunscreen or photoprotective measure. However, recent research has clearly demonstrated that sub- Minimal Erythemal Doses (MED) doses of UV radiation, or even longer wavelengths can also have profound effects on the skin (5). These effects range from adaptive responses such as increased melanin production (6) to skin damage. This is particularly true for UVB sub-MED, which may cause cancer (7) and local immunosuppression (8, 9), even at very low (<15% MED) doses (10, 11).

Given that immunosuppression is one of the hallmarks of cancer (12), it is possible that a sunscreen that displays excellent SPF may not prevent photocarcinogenesis due to the combination of subMED skin damage including oxidative stress and immunosuppression, particularly in cancer-prone individuals. Poon et al. (11) demonstrated that prevention of immunosuppression by sunscreens in humans is not related to the MED, as this parameter depends much more strongly on UVB than UVA. This suggests that MED measurements (the basis for SPF determination) do not accurately estimate the dose of UV that may cause immunosuppression. This makes it necessary to widen the type of measurements to ensure that novel formulations exert more biological effects, thereby preventing photoimmunosupression. De Fabo and Noonan described that skin immunosuppression in terms of inhibition of contact hypersensitivity (CHS) in mice depends on the applied wavelength, with a peak between 260 and 290 nm and declining until 320 nm (13). This was done using contact irritants, 2-chloro-l,3,5-trinitrobenzene (TNCB) or 1-fluoro-2,4-dinitrobenzene (DNFB), in the presence of UV light in a murine model (14). The irritants were applied on the ear, then UV of different wavelengths and intensities were applied, and ear swelling was measured. Swelling was a proxy for inflammation, which is a mark of an efficient immune response, and used to determine the UV action spectra at different wavelengths. More recently, another study described that UV radiation induces immunosuppressive effects in human skin using *in vivo* analysis of the nickel model of recall contact hypersensitivity, which works in a similar manner as CHS, but uses nickel as the irritant. Again, swelling is used as a mark of an efficient contact response that is decreased by UV light. In this work, two major bands were identified, one at 300 nm (UVB) and another around 370 nm (UVA) (15). The latter is more pertinent when discussing immunosuppression, so due to the highest solar UVA radiation reaching the earth surface, it can be explained the broadband UV dependence of immunosuppression due to the combined effect of UVA together to UVB. Thus, the assay described above was the basis of the human immune protection factor (HIF) used here. Based on these and other lines of evidence, there is a general trend toward the development of sunscreens containing natural components that may act as physical sunscreens while also providing a biological role as antioxidant or immunomodulator, alone or in combination with chemical sunscreens of proven efficacy to decrease erythema.

Fernblock® (from here on referred to as PLE) is a hydrophilic natural extract from *Polypodium leucotomos* with proven efficacy over other extracts of the same fern due to the extraction method (16). It has been extensively studied in photobiology of the skin due to is due to its antioxidant properties against reactive oxygen species production induced by UV radiation, protective activity to DNA damage, and prevention of UV-mediated apoptosis, necrosis and degradative matrix remodeling as well as acting as a potent immunomodulator [reviewed in Parrado et al. (17)]. The presence of a high percentage of phenolics (mainly benzoates and cinnamates, like caffeic acid and its derivative ferulic acid) confers also UV absorption properties of PLE (18), PLE exerts a dual role on skin, acting as a biological agent with active properties and as a sunscreen.

This work aims to analyze the absorption properties of PLE and its combination with organic and mineral sunscreens to enhance the sunscreen capability of the organic and mineral component of the formulation, and whether its inclusion in galenic formulations boosts immunoprotective parameters used as ISO standards.

## Materials and Methods

### PLE Formulation

Fernblock®, PLE, is a controlled hydrophilic extract from the leaves of *P. leucotomos* (16). PLE was provided as lyophilized powder by Cantabria Labs, Madrid, Spain. The powder was stored at room temperature shielded from light following the supplier's instructions. Stock solutions were prepared at a concentration of 6.25, 12.5, 25, and 50 μg/ml mg/ml in distilled water.

### Preparation of Sunscreen Formulation

PLE extract was included in four experimental galenic formulations similar to those used in sunscreen formulations, containing different types of UVB and UVA organic and mineral filters together with PLE at 1% (Table 1). For each full sunscreen formula, three different compositions were assayed in each case: (1) PLE alone; (2) Filters; (3) Full sunscreen: PLE + filters.

**Table 1:** Different combinations of UVB and UVA organic and mineral filters used to prepare the experimental sunscreens used throughout the study.

**SAMPLE 1**	Ethylhexyl Salicylate, Octocrylene, Butyl Methoxydibenzoylmethane, Ethylhexyl Triazone, Diethylamino Hydroxybenzoyl Hexyl Benzoate, Phenylbenzimidazole, Sulfonic Acid, Tris-Biphenyl Triazine (nano), Decyl Glucoside, Butylene Glycol, Disodium Phosphate, Xanthan Gum, Aqua.
**SAMPLE 2**	Phenylbenzimidazole, Sulfonic Acid, Disodium Phenyl Dibenzimidazole Tetrasulfonate, Octocrylene, Butyl Methoxydibenzoylmethane, Bis-Ethylhexyloxyphenol Methoxyphenyl Triazine, Bis-Ethylhexyloxyphenol Methoxyphenyl Triazine, Cyclopentasiloxane, Titanium Dioxide (nano), Polyglyceryl-3 Polydimethylsiloxyethyl Dimethicone, Aluminum Hidroxide, Stearic Acid, Tris-Biphenyl Triazine (nano), Decyl Glucoside, Butylene Glycol, Disodium Phosphate, Xanthan Gum, Aqua.
**SAMPLE 3**	Ethylhexyl Salicylate, Ethylhexyl Triazone, Bis-Ethylhexyloxyphenol Methoxyphenyl Triazine, Diethylamino Hydroxybenzoyl Hexyl Benzoate, Cyclopentasiloxane, Titanium Dioxide (nano), Polyglyceryl-3 Polydimethylsiloxyethyl Dimethicone, Aluminum Hidroxide, Stearic Acid, Zinc Oxide (nano), Triethoxycaprylylsilane, Tris-Biphenyl Triazine (nano), Decyl Glucoside, Butylene Glycol, Disodium Phosphate, Xanthan Gum, Aqua.
**SAMPLE 4**	Ethylhexyl Methoxycinnamate, Octocrylene, Diethylamino Hydroxybenzoyl Hexyl Benzoate, Butyl Methoxydibenzoylmethane, Ethylhexyl Triazone, Zinc Oxide (nano), Triethoxycaprylylsilane, Titanium Dioxide (nano), Alumina, Simethicone, Aqua.

*PLE was analyzed including a concentration of 1% of PLE in the four formulations*.

### Absorbance Properties of PLE Analysis

To analyze the potential of PLE as sunscreen, four different concentrations of PLE (6.25, 12.5, 25, and 50 μg/ml) were diluted in distilled water under constant stirring at 25–30°C and their absorbance in the UV-visible (250–700 nm) were measured in quartz UV-transparent cuvette in a UV-visible spectrophotometer Shimazdu UV-1607 (Shimazdu Co., Kioto, Japan).

### Protection Factors of Sunscreen Formulations

The spectral transmittance of the different formula containing only PLE, filters or full sunscreen were calculated as well as the spectral absorbance of them. Absorbance was calculated for each wavelength in the interval of 290–400 nm following the formula:

$$\begin{array}{l} \text{Absorbance}=-Log\left(\text{Transmittance}\right) \end{array}$$

The protection factor of each formulation were calculated *in vitro* by measuring the spectral transmittance of formulas in the UV range (290–400 nm) in PMMA plaques (Schönberg, Hamburg, Germany), following the protocol indicated in ISO 24443:2012 for the analysis of the UVA protection factor for sunscreens (19).

Briefly, transmittance spectra was determined by evenly spreading 1.3 mg/cm^2^ of the product over a 5 × 5 cm^2^ PMMA plate. The plate had a roughness simulating that of real skin relief, as indicated by the aforementioned ISO regulation. After 15 min in the darkness, the sample was placed on the sensor (Ulbrich sphere type) of a Macam SR-2210 double monochromator spectroradiometer (Macam, Scotland), and illuminated with a 300 W Oriel solar simulator (Oriel, Newport Corporation, Irvine, US). Spectral transmittance spectrum was analyzed at 1 nm intervals in the range 290–400 nm, referred to the spectral transmittance of the blank PMMA plate coated with glycerol.

Sun protection factor (SPF) was calculated as the protection potential against skin erythema (14) using the following formula:

$$\begin{array}{l} SPF=\;\frac{\int_{290}^{400}\left(E_{\lambda }\times \;\varepsilon_{\lambda }\right)}{\int_{290}^{400}\left(E_{\lambda }\;\times \;\varepsilon_{\lambda }\;\times \;T_{\lambda }\right)} \end{array}$$

In which SPF, sun protection factor; E, spectral irradiance of solar simulator; ε, relative effectiveness for erythema; T, Transmittance of the sample.

**Figure 1:**
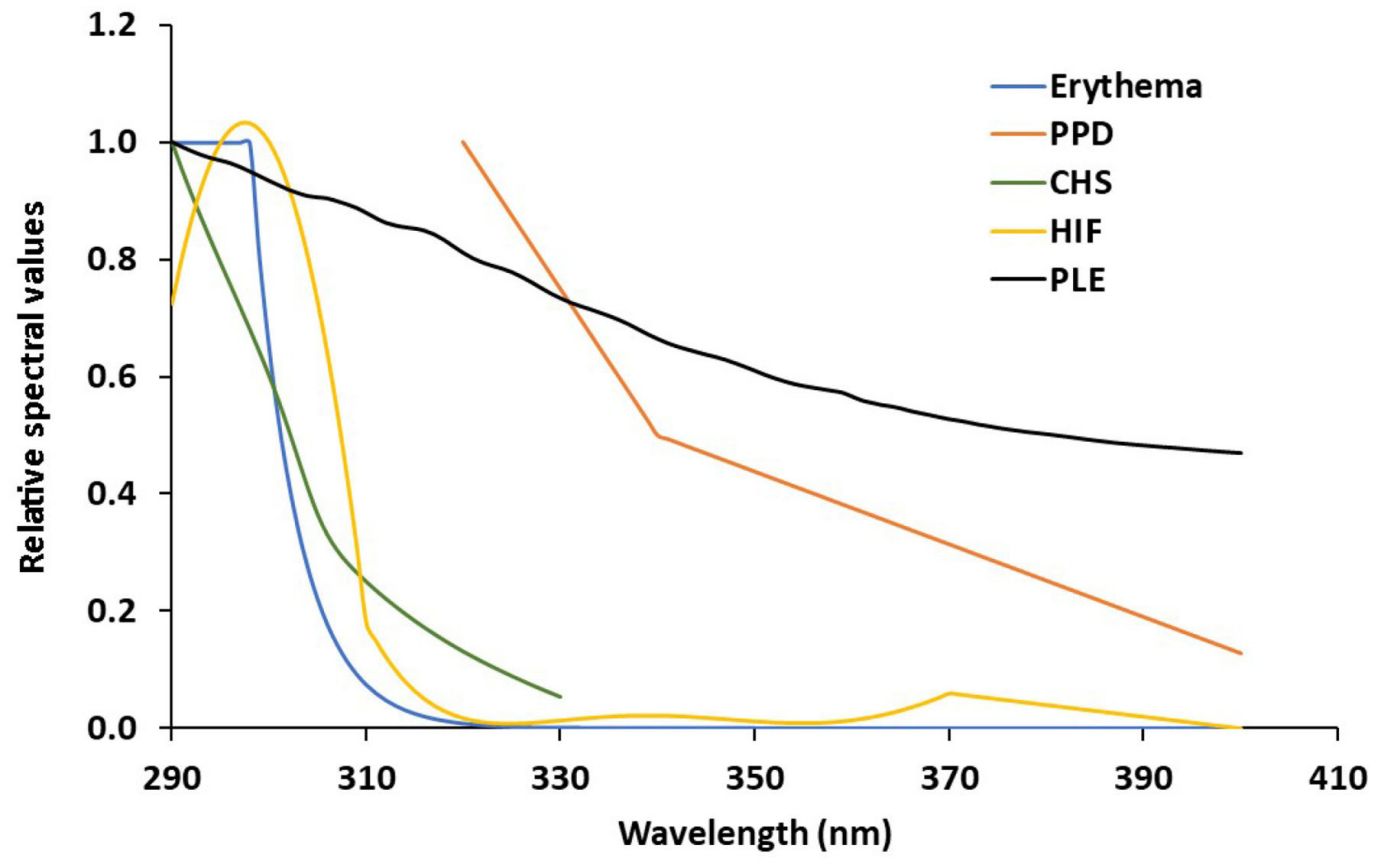
Spectral absorbance of PLE related to the different action spectra analyzed (Erythema; PPD, Persistent Pigment Darkening; CHS, Contact Hypersensitivity factor; HIF, Human Immunoprotection Factor; PLE, *Polypodium leucotomos* extract, Fernblock®).

UVA protection factor was also calculated by determining the action spectrum of Persistent Pigment Darkening as described in ISO 24443:2012. To determine protection against photo immunosuppression, sample transmittance in the UV region was pondered by the action spectra published for the contact hypersensitivity (14), and human skin photoimmunosuppression (15). The action spectra data was analyzed at 1 nm intervals in the range 290–400 nm from cubic spline interpolation between the data points of the respective action spectrum to provide values of 1 nm increments. The integral in the equation was replaced by the sum of the data obtained at each step of 1 nm. Spline interpolation was carried out using Table curve 2D 5.0. Error in the interpolation and 1 nm-step data sum is estimated to be <5%. The action spectrum of erythema, Persistent Pigment Darkening, contact hypersensitivity, and human skin photoimmunesupression are shown, compared to the absorbance of aqueous extracts (50 μg/ml) of PLE are shown in Figure 1.

Critical wavelength was also determined. Critical wavelength defines the performance of a sunscreen in the whole UV solar spectrum and it is identified as the upper limit of the spectral range from 290 nm on, covering 90% of the area under the extinction curve of the whole UV range between 290 and 400 nm. When the critical wavelength is 370 nm or greater, the product is considered broad spectrum, which denotes balanced protection throughout the UVB and UVA ranges.

### Statistics

Data regarding Protection Factor for different UV skin biological effects (erythema, PPD, CHS and HIF) as well as critical wavelength, based on UV transmittance was determined in three different places of 25 cm^2^-PMMA plaques. Three plaques were used for each treatment (glycerol, base formula + PLE extract 1% and full formula with combination of PLE with sunscreens). Protection factors are determined using a total of nine sub-replicates. From the nine replicates, the mean ± SD was calculated. In order to accept the final protection factor with this number of replicates, the confidence interval of 95% had to be lower than 17% with respect to the mean value. Booster effects have been analyzed in terms of % of change of biological factors between the full formulations compared to PLE 1% alone in base formula. Comparison of the mean protection factors between PLE alone with respect to the full sunscreen formula has been made using Student's *t*-test. Significance was considered ≤0.05 as per the standard of the field. Statistics were performed using 2019 Excel Program.

## Results

### UV Absorbance of PLE

**Figure 2:**
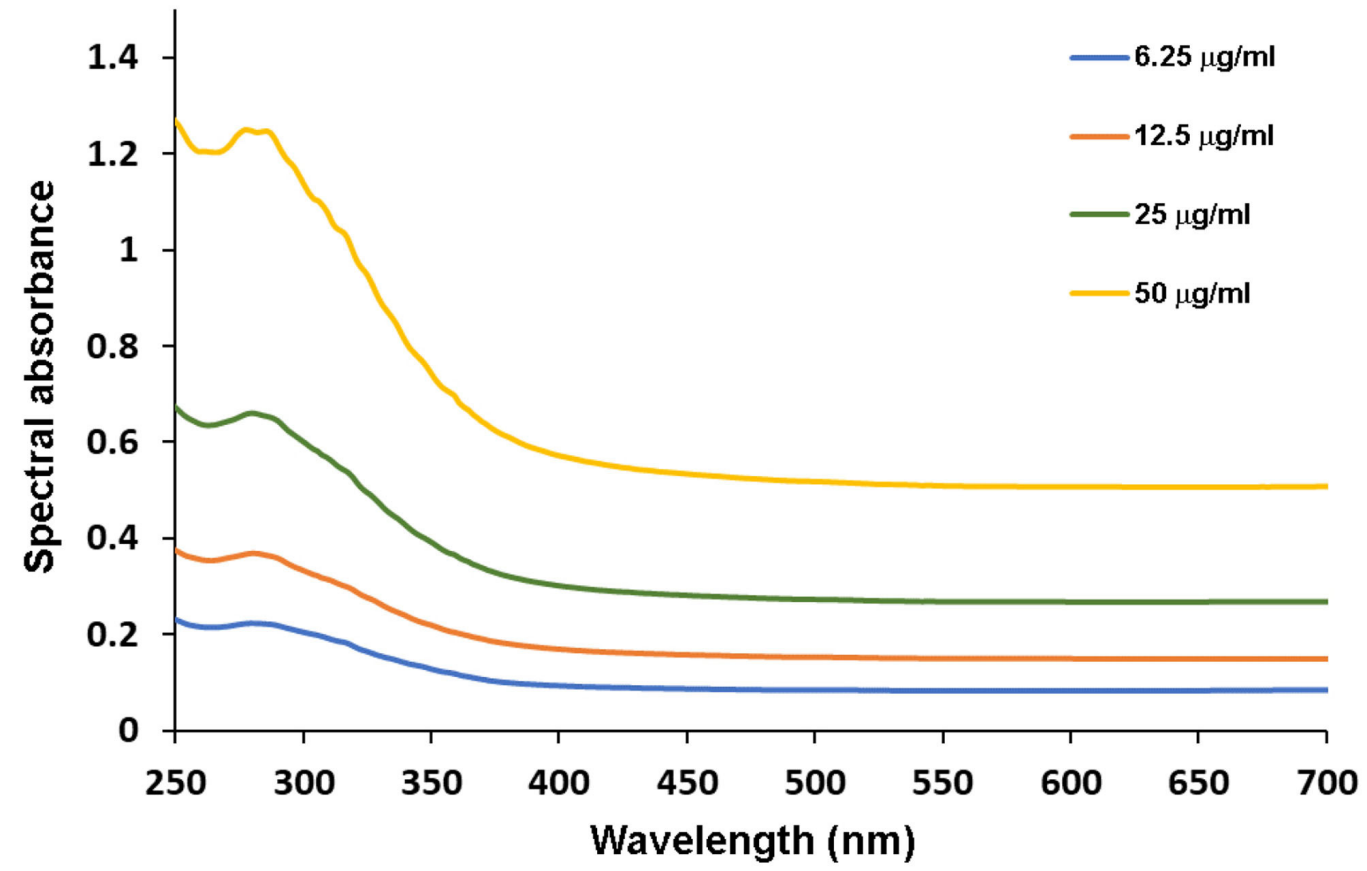
Spectral absorbance in the UV and visible spectral regions (250–700 nm) of different concentrations of the PLE extract diluted in distilled water at different concentrations (6.25, 12.5, 25, and 50 μg/ml). Data is representative of three independent experiments made in triplicates.

The different concentrations of PLE diluted in distilled water increased absorbance in the UV spectrum, gradually from 250 to 400 nm, reaching a peak around 290 nm (Figure 2). Due to the brownish color of the different concentrations of PLE extract in water, their absorbance in the visible region also increased, with values reaching 0.6 absorbance units along the entire visible spectrum (400–750 nm; Figure 2).

### PLE Booster Effect in Different Sunscreen Galenic Formulations

**Figure 3:**
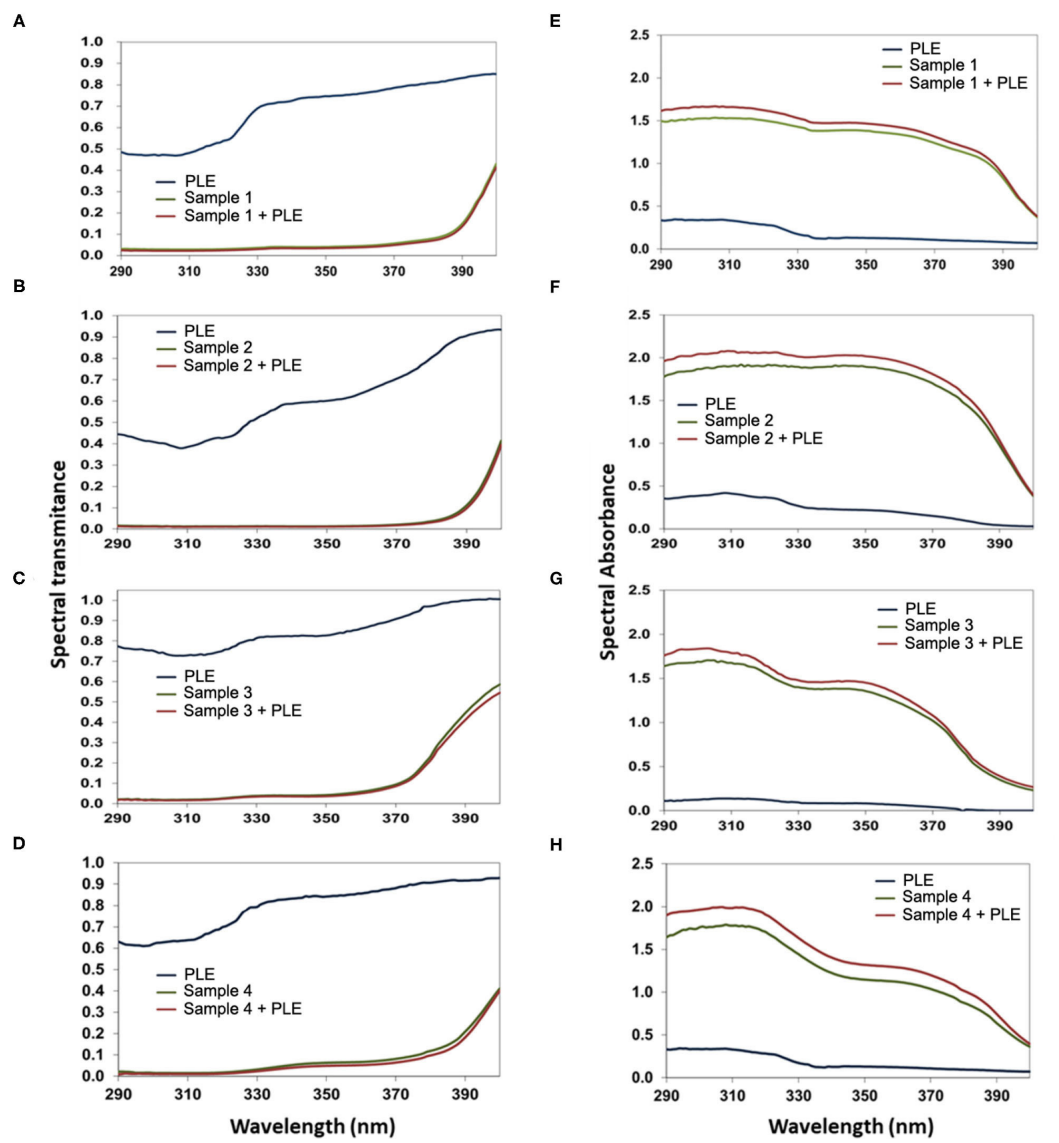
Spectral transmittance **(A–D)** and absorbance **(E–H)** of PLE alone, sample 1 **(A,E)**, 2 **(B,F)**, 3 **(C,G)**, and 4 **(D,H)** without PLE, or a combination of both compared to the formula containing (Sample 1–4) only filters and the formula containing only PLE (Sample 1–4+PLE). Please refer to the Materials and Methods section and Table 1 for details on the formulations used in each case.

The booster effect of PLE in galenic formulations of full sunscreens is shown in Figure 3. 1% PLE alone (in the same excipient formula as that of full sunscreen) displayed a gradual decrease in UV transmittance from 290 to 400 nm, reaching the bottom value at ≈310 nm (Figures 3A–D). The combinations of filters alone significantly decreased UV transmittance up to 400 nm, with a wider range of low values from 290 to 390 nm. The booster effect of PLE is clearly observed when absorbance is analyzed for all four different combinations of UV filters (Figures 3E–H). One percent of PLE alone (in the same excipient formula as that of full sunscreen) displayed an absorbance peak at 308 nm of ≈0.4 absorbance units in the different galenic formulas. When PLE was combined with the UV filters, the absorbance curve was significantly enhanced in all cases, leading to absorbances >2 as shown in Figures 3E–H.

Next, we used the transmittance curves to calculate the protection factor by ponderation with the different action spectra. Results are shown in Table 2. PLE markedly increased SPF in the different formulas. In case of formulation 1, thought the PLE alone has a SPF of 2.52 when PLE is included in the final formulation increased SPF from 37.99 to 42.22. In case of sample 3, PLE showed a SPF of 1.55 but in this case, when it is combined with filters, SPF is increased over 20%. So, the average booster effect in SPF obtained from the four different combinations was 14.16% (Table 2).

**Table 2:** Solar protection factors, UVA protections factors, the relation between UVA/UVB, the critical wavelength (CW), the contact hypersensitivity factor (CHS), and the human immunoprotection factor (HIF) for different combinations of filters with PLE (full sunscreen) compared with the formula containing only filters and the formula containing only PLE.

**SAMPLES**		**SPF**	**CHS**	**HIF**	**UVAPF**	**CW**
**Sample 1**	**Filters**	37.99 ± 3.58	38.91 ± 3.88	27.44 ± 3.4	18.82 ± 2.72	383 ± 0.15
	**PLE**	2.52 ± 0.10	2.37 ± 0.017	1.90 ± 0.12	1.63 ± 0.17	380 ± 0.30
	**Full sunscreen**	42.22 ± 5.12	42.95 ± 5.28	30.09 ± 2.73	20.68 ± 1.23	383 ± 0.21
	**Boost (%)**	**11.13**	**10.38**	**9.65**	**9.88**	**–**
**Sample 2**	**Filters**	67.17 ± 9.44	71.03 ± 10.81	51.23 ± 5.14	30.09 ± 2.71	383 ± 0.20
	**PLE**	2.36 ± 0.06	2.35 ± 0.06	1.79 ± 0.15	1.52 ± 0.19	371 ± 0.22
	**Full sunscreen**	75.62 ± 9.55	82.84 ± 7.54	60.23 ± 6.15	32.38 ± 2.21	382 ± 0.18
	**Boost (%)**	**12.58**	**16.62**	**17.56**	**7.61**	**–**
**Sample 3**	**Filters**	38.53 ± 3.07	39.43 ± 3.78	15.79 ± 1.28	8.52 ± 0.31	376 ± 0.25
	**PLE**	1.55 ± 0.05	1.51 ± 0.05	1.53 ± 0.04	1.42 ± 0.01	375 ± 0.21
	**Full sunscreen**	46.49 ± 3.53	47.71 ± 3.63	17.49 ± 1.36	9.44 ± 0.44	378 ± 0.01
	**Boost (%)**	**20.66**	**21.00**	**10.77**	**10.80**	**–**
**Sample 4**	**Filters**	66.85 ± 6.15	70.72 ± 3.21	25.85 ± 2.61	15.78 ± 1.27	378 ± 0.31
	**PLE**	1.48 ± 0.01	1.48 ± 0.01	1.56 ± 0.02	1.48 ± 0.03	378 ± 0.30
	**Full sunscreen**	75.05 ± 10.79	79.18 ± 11.71	27.10 ± 3.47	17.21 ± 1.97	378 ± 0.22
	**Boost (%)**	**12.26**	**11.96**	**4.83**	**9.06**	**–**
**Average boost (%)**		**14.16**	**14.99**	**10.70**	**9.34**	**–**

When we estimated UVA-PF, the enhancer effect of PLE in full sunscreen was lower than that obtained for SPF, but still significant, with a medium UVA-PF increase of 9.34%. This is consistent with the lower absorbance of PLE in this region of the light spectrum. Nevertheless, all the formulas analyzed showed critical wavelengths over 370 nm. Thus, PLE maintains the typical broad spectrum of these sunscreens formulas.

### PLE Boosts Photo Immunoprotection-Related Action Spectra

We next examined the ability of these preparations to prevent photoimmunosuppression. To do this, we analyzed two different action spectra. First, we estimated its effect on CHS (14). CHS photoprotection displayed by the four different formulas was quite similar to that of SPF; the addition of PLE to the formula led to increased CHS protection factor (14.99%), suggesting that the booster effect of PLE in CHS is comparable to that of SPF. We also estimated the HIF index, which has a higher contribution of UVA wavelengths than that of erythema and CHS (15). Thus, we found an improvement degree of protection in the sunscreen combinations, though less than the CHS index. The enhancer effect of PLE was lower compared to the other biological effects. UVA absorption of the product allows us to predict a mean enhancer effect ≈9.34% (Table 2).

## Discussion

The present study demonstrates that PLE has broad absorption spectrum with a gradual increase up to 290 nm that correlates with that of the erythematous spectrum. It also correlates well with photoimmunoprotection spectra at different UV wavelengths. The fact that PLE absorbs UV photons by itself (Figure 1) allows us to predict that it will display broadband protection along the UV spectrum, although this is likely to be more significant at UVB wavelengths. A concentration of 1% PLE alone in the formula leads to a mean SPF, CHS, and HIF around 2, which could be considered as a booster effect. Strikingly, the addition of PLE to different combinations of organic and mineral sunscreens has a booster effect with a mean increase of SPF, CHS and HIF factor over 10 arbitrary units (sample 2, Table 2) and more than 10% of average boost of all factors.

Use of natural products in cosmetics is a current trend; thus, the discovery of new UV natural absorbing compounds will reduce need for high concentrations of organic chemical sunscreens in formula and reinforce the biological protection. This is important as some organic components used may have deleterious effects on both humans and the environment. Also, the reduction of these kinds of ingredients improves the galenic formulations and consequently could enhance the photoprotection adherence. Other natural compounds similar to the PLE extract used here may function as UV filters against induced damage in keratinocytes (20); some isoflavones, like genistein and daidzein, also block UVB induced skin burns in human and provide protection against photocarcinogenesis and photoaging (21). Other natural sunscreens are mycosporine-like amino acids synthetized by marine algae, fungi, and lichens. The compounds are endowed with extremely high UVB/UVA extinction coefficients and display negligible toxicity, high photo-stability and antioxidant properties (22–24).

The significant barrier activity of PLE complements the current state of the art of this compound, which is mainly related to photoprotection in terms of erythema, DNA protection and permanent pigmentation darkening (PPD). The data contained herein strongly suggests that it provides an additional layer of protection by curbing photoimmunosuppression. Validation of the evaluation of action spectra to provide relevant biological information also suggests the potential immunoprotective usefulness of other biological sunscreens, e.g., mycosporine-like amino acids (25). The overarching concept is to incorporate these biologically active natural sunscreens to a global strategy that includes oral photoimmunoprotection and use of multi-functional sunscreens.

In this regard, Schalka and Donato recently reported that the PLE incorporation to sunscreens markedly decreased UV-mediated sunburn and CD1a+ depletion in human volunteers (26). Although this work did not analyze the absorption of PLE across the skin, the data confirms the potentially beneficial effect of incorporating PLE into sunscreen formulations in order to reduce the clinical and biological deleterious effects caused by cutaneous exposure to solar radiation.

In all, the enhancer effect of PLE and its ability to boost both erythemal and photo-immunoprotection potential of conventional sunscreens confirms the data obtained using orally ingested PLE. It is important to highlight that immunosuppression, although more severe at UVB wavelengths in *in vitro* settings, is actually more relevant at UVA wavelengths, due to the fact that many more UVA photons reach the surface of the Earth (15). This is the main difference between the CHS measurements derived from the data published in De Fabo and Noonan (14), referred here as CHS; and the findings of Damian et al. (15), which form the basis of the HIF index. CHS was determined in the 250–320 nm range, which is UVB and correlates with erythema. Conversely, HIF includes the contributions of UVB (in this part of the UV spectrum, it is indeed comparable to CHS), but also UVA, which is likely more significant for immunosuppression despite inducing much less erythema than UVB.

Even at lower effective concentrations, PLE has a positive effect that predicts not only its efficacy as a sunscreen, but also has biological value. *In vitro*, PLE protects human skin cells subjected to UV irradiation (27). Such protection extends to dendritic cells (28). Importantly, *trans*-urocanic acid isomerization to the *cis* form as been proposed as a crucial feature of immunosuppression not only by UVB photons, but also by UVA photons in the presence of psoralens (29). In good agreement with its photoimmunoprotective effect, PLE decreases trans-UCA isomerization (30). It is feasible that PLE absorbs some of the deleterious UV photons *in situ*, while providing positive feedback signals that protect immune cells, contributing to the photo immunoprotective effect described here.

**Figure 4:**
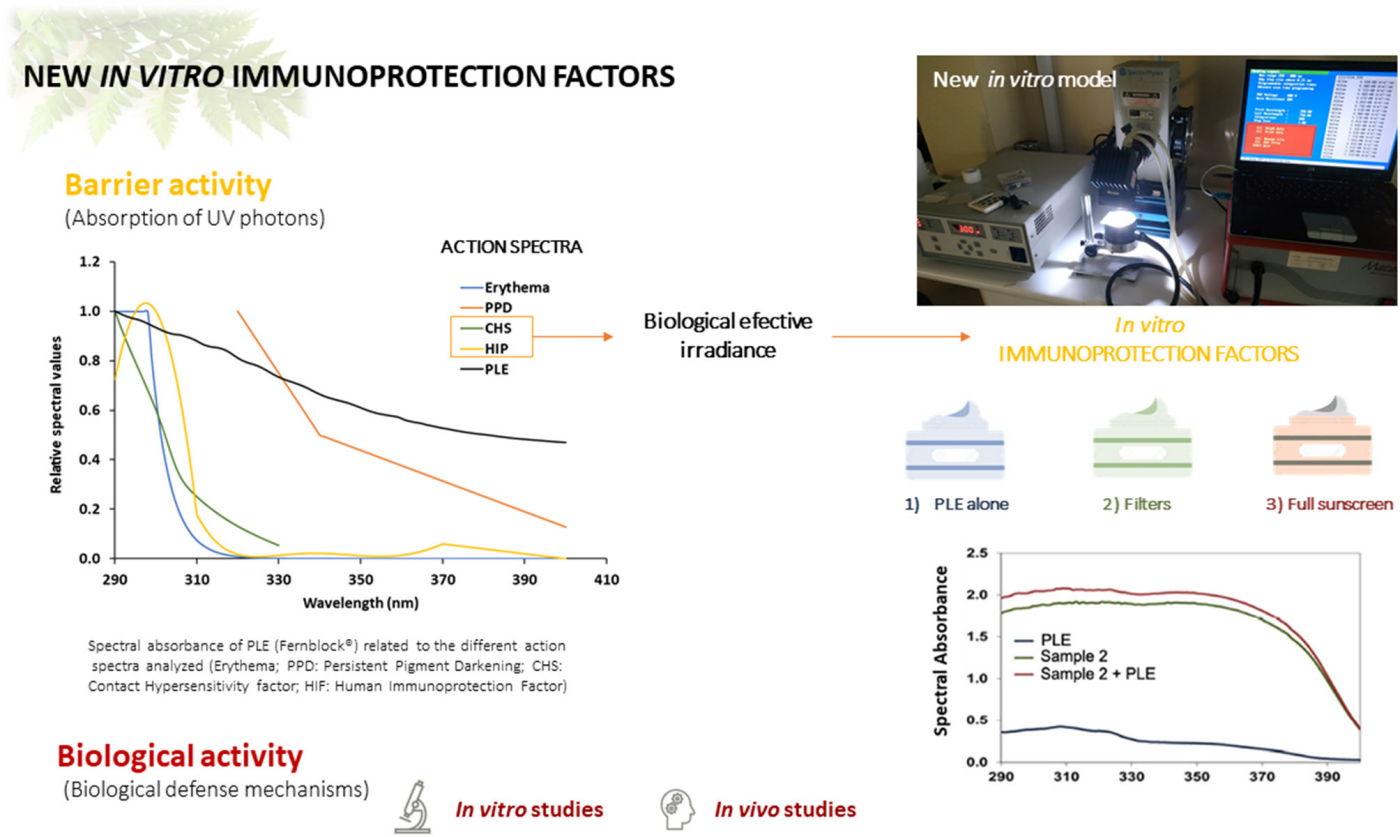
Summary of the effect of PLE (Fernblock®) on diverse action spectra related to erythematous photoprotection and photoimmunoprotection. PLE is endowed with both types of activity when incorporated to canonical sunscreen formulations.

Taken together with the evidence of oral photoprotection displayed by PLE, the data herein suggest a paradigm change in which physical sunscreens, while efficient, would not be sufficient. Indeed, some evidence indicates that photoaging and photo immunosuppression are not sufficiently curbed by physical photon blockers due to a strong influence of UVA photons in the generation of these biological effects. New generation sunscreens need to promote additional effects, not only with filters, but with compounds that promote both regeneration and/or immunoprotection. Evidently, more research in human patients is needed to complete the assessment of this PLE for incorporation in topical sunscreen formulations, but this early evidence indicates that this could be a mechanism to promote additional beneficial effects, leading to a multi-pronged protection network that includes barrier/photon blocking function as well as anti-inflammatory, anti-aging and immunoprotective biological activity (Figure 4).

## Data Availability Statement

The original contributions presented in the study are included in the article/supplementary material, further inquiries can be directed to the corresponding author/s.

## Author Contributions

SG, JA, and AR-L: conceptualization. JA, MG, and EH-C: methodology, investigation, and resources. JA: software, data curation, and formal analysis. SG, MG, EH-C, and AR-L: validation. MV-M, JA, SG, and AR-L: writing—original draft preparation and writing—review and editing. MG, EH-C, SG, and AR-L: supervision. AR-L: project administration. AR-L and SG: funding acquisition. All authors have read and agreed to the published version of the manuscript.

## Conflict of Interest

AR-L belongs to the Innovation and Development Department of Cantabria Labs, which produces Fernblock^®^. SG is a consultant for Cantabria Labs. The remaining authors declare that the research was conducted in the absence of any commercial or financial relationships that could be construed as a potential conflict of interest.

## Abbreviations

MED: minimal erythematous dose
UV: ultraviolet
PLE: *Polypodium leucotomos* extract
SPF: sun protection factor
UVA-PF: ultraviolet-A protection factor
LC: Langerhans cells
PMMA: Polymethylmetacrilate
CHS: Contact HiperSensitivity
HIF: Human Immunoprotection Factor
PUVA: psoralens-UVA.
